# The Prognostic Role of Early Skeletal Muscle Mass Depletion in Multimodality Management of Patients with Advanced Gastric Cancer Treated with First Line Chemotherapy: A Pilot Experience from Modena Cancer Center

**DOI:** 10.3390/jcm10081705

**Published:** 2021-04-15

**Authors:** Margherita Rimini, Annarita Pecchi, Francesco Prampolini, Chiara Bussei, Massimiliano Salati, Daniela Forni, Francesca Martelli, Filippo Valoriani, Fabio Canino, Alessandro Bocconi, Fabio Gelsomino, Linda Reverberi, Stefania Benatti, Federico Piacentini, Renata Menozzi, Massimo Dominici, Gabriele Luppi, Andrea Spallanzani

**Affiliations:** 1 Division of Oncology, Department of Oncology and Hematology, University Hospital of Modena, 41122 Modena, Italy; margherita.rimini@gmail.com (M.R.); massisalati@gmail.com (M.S.); fabiocanino05@gmail.com (F.C.); alessandro.bocconi@gmail.com (A.B.); fabiogelsomino83@yahoo.it (F.G.); stefania.benatti@unimore.it (S.B.); federico.piacentini@unimore.it (F.P.); massimo.dominici@unimore.it (M.D.); luppi.gabriele@aou.mo.it (G.L.); 2 Department of Radiology, University Hospital of Modena, 41122 Modena, Italy; pecchi.annarita@aou.mo.it (A.P.); prampolini.francesco@aou.mo.it (F.P.); danielaforni92@gmail.com (D.F.); francesca.martelli1992@gmail.com (F.M.); 3 Division of Metabolic Disease and Clinical Nutrition, University Hospital of Modena, 41122 Modena, Italy; bussei.chiara@aou.mo.it (C.B.); valorianifilippo@gmail.com (F.V.); reverberi.linda@policlinico.mo.it (L.R.); menozzi.renata@aou.mo.it (R.M.)

**Keywords:** gastric cancer, sarcopenia, nutritional status

## Abstract

Background: Few data about the link between nutritional status and survival are available in the metastatic gastric cancer (GC) setting. The aim of this work was to evaluate the prognostic role of tissue modifications during treatment and the benefit of a scheduled nutritional assessment in this setting. Methods: Clinical and laboratory variables of 40 metastatic GC patients treated at Modena Cancer Center were retrieved: 20 received a nutritional assessment on the oncology’s discretion, the other 20 received a scheduled nutritional assessment at baseline and every 2–4 weeks. Anthropometric parameters were calculated on Computed Tomography (CT) images at the baseline and after 3 months of chemotherapy. Results: A correlation between baseline Eastern Cooperative Oncology Group Performance Status (ECOG PS), Lymphocyte to Monocyte Ratio (LMR), C-reactive protein (PCR), Prognostic Nutritional Index (PNI) and Overall survival (OS) was highlighted. Among the anthropometric parameters, early skeletal muscle mass depletion (ESMMD) >10% in the first months of treatment significantly impacted on mOS (*p* = 0.0023). A link between ESMMD and baseline LDH > 460 U/L, baseline CRP > 2.2 mg/dL and weight decrease during treatment emerged. Patients evaluated with a nutritional scheduled support experienced a mean gain in subcutaneous and visceral fat of 11.4% and 10.21%, respectively. Conclusion: We confirm the prognostic impact of ESMMD > 10% during chemotherapy in metastatic GC. The prognostic role of a scheduled nutritional assessment deserves further confirmation in large prospective trials.

## 1. Introduction

The prognosis of patients with advanced gastric cancer is still poor due to the absence of potentially curative options [1]. Palliative chemotherapy improves survival and quality of life (QoL) compared to best supportive care both in first and second line setting [2]. In recent years, the development of new drugs alone or in combination with chemotherapy, helped to raise the bar of median overall survival over 12 months at the expense of increased treatment related toxicities [3,4]. The introduction of anti-HER2 treatment in first such as the development of ramucirumab alone or in combination with chemotherapy in second line provided nearly 14–16 months of median overall survival in patients with new diagnosis of metastatic gastric cancer [5,6,7]. In fact, alongside progress in the pharmacological field, the target has moved to an adequate patient selection. The research of clinic-pathological prognostic and predictive factors is one of the main objectives of prospective studies and retrospective analysis [8,9].

Some prognostic scores have been developed combining inflammation-related and nutrition-related markers, such as the neutrophil-to-lymphocyte ratio (NLR) platelets to Lymphocyte ratio (PLR), lymphocyte to monocyte ratio (LMR) and prognostic nutritional index (PNI): the prognostic role of these parameters and other factors such as performance status and neoplastic markers (CEA and CA 19.9) is well known in patients with advanced gastric cancer [10,11].

Almost two decades ago it was identified the close link between malnutrition and survival and the impact of sarcopenia on tolerance to chemotherapy, longer hospitalization, quality of life and mortality, but to date, many malnourished cancer patients still receive inadequate nutritional support, mainly due to the poor awareness of the problem and inefficient collaboration between oncologists and clinical nutritionists [12].

An adequate evaluation of patients’ nutritional status in metastatic setting cannot ignore the assessment of skeletal muscle mass and skeletal muscle density using computed tomography scan. Previous studies in various types of cancer highlighted the strong association between survival, treatment toxicities and the amount of muscle and adipose tissue at diagnosis such as its modification during chemotherapy [13,14,15,16].

A recent metanalysis focused on the effects of dietary interventions on nutritional status of gastric cancer patients undergoing gastrectomy, but few data are available in patients with metastatic gastric cancer treated with palliative chemotherapy [17].

The aim of our work is to evaluate the incidence of sarcopenia such as the prognostic and predictive role of muscle and visceral tissue modifications during the first 3 months of chemotherapy in patients with metastatic gastric cancer. In a subsequent pivotal analysis, we matched two different group of patients to evaluate the role of an adequate nutritional support during palliative chemotherapy.

## 2. Materials and Methods

Patients with recurrent or metastatic gastric cancer who received fluoropyrimidines and platinum based first-line chemotherapy in Modena Cancer Center from November 2015 through December 2019 were retrospectively studied. All patients had histologically proven adenocarcinoma of stomach with at least one metastatic lesion as confirmed by diagnostic imaging. Computed Tomography (CT) scan was performed every 2–4 months in most patients to evaluate treatment efficacy. The study was approved by local Ethic Committee (n° 427/2019/OSS/AOUMO). All alive patients provided written informed consent.

Clinical and laboratory data were reported from the hospital electronic medical database at diagnosis and first CT re-evaluation including the following variables: age, gender, performance status (ECOG), height, weight, Body-Mass Index (BMI), blood count, neutrophil/lymphocyte ratio (NLR), platelets/lymphocyte ratio (PLR), lymphocyte/monocyte ratio (LMR), systemic inflammatory index (SII) lactate dehydrogenase (LDH), C-reactive protein (CRP), albumin, Sodium (Na^+^), Potassium (K^+^), CEA, CA 19.9 and prognostic nutritional index (PNI).

In the second part of the work, we searched for differences in terms of clinical, anthropometric and survival outcomes between the first group of patients which received a nutritional evaluation at oncology’s discretion and the second group of patients which received a standardized nutritional evaluation at the baseline and then every 2–4 weeks during treatment.

The nutritional evaluation was defined as an individualized counselling aimed to collecting data about the dietary intake, usual dietary pattern, intolerances or food aversions, digestive difficulties, patients’ psychological status, autonomy and need for help in the act of eating. In addition, a symptom assessment was included in the nutritional evaluation. Each nutritional assessment resulted in dietary advice and, if necessary, in prescription of oral implementations.

### 2.1. Body Composition Parameter Measurements

All patients included in the study underwent CT scan at the time of diagnosis and after 2–4 months, as part of the diagnostic and therapeutic path planned by the cancer center.

CT exams were performed at our hospital using a 64-slice CT scanner (Lightspeed VCT, GE Healthcare, Milwaukee, WI, USA).

Baseline and follow-up CT examinations were loaded on an Advantage Workstation (VolumeShare 7, GE Healthcare, Milwaukee, WI, USA) and non-contrast images at the level of the third lumbar vertebra (L3) were used for reconstructions and measurements of quantitative and qualitative body composition parameters.

**Figure 1:**
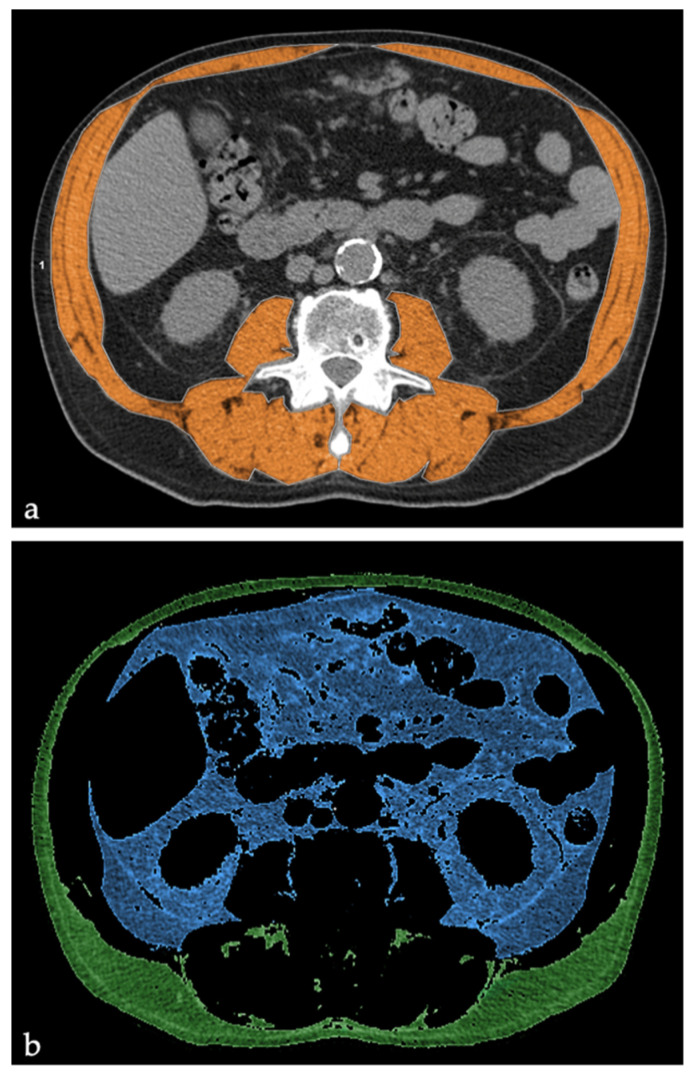
Measurement of body composition parameters with cross-sectional computed tomography (CT) images at the level of third lumbar vertebra. (**a**) Skeletal muscle area (**b**) Subcutaneous and Visceral fat areas.

According to literature, skeletal muscle cross-sectional areas including the psoas, paraspinal muscles and abdominal muscles were identified and quantified using the preestablished HU thresholds for muscle (HU-30 to 150), whereas subcutaneous and visceral adipose cross-sectional areas were quantified using HU thresholds for fat tissue (HU-150 to-30) (Figure 1).

The skeletal muscle mass index (SMI, cm^2^/m^2^) was calculated by dividing these skeletal muscle areas by height squared and similarly visceral fat index (VFI, cm^2^/m^2^) and subcutaneous fat index (SFI, cm^2^/m^2^) were calculated by normalizing each fat area for height.

Total adipose index (TAI) was calculated by adding SFI + VFI.

Relative changes of body composition occurred in the period between baseline and follow-up CT scans were also quantified for each patient by calculating delta (Δ) parameters: ΔSMI (defined also as ESMMD), ΔSFI, ΔVFI, ΔTAI. SMI reduction between baseline and first evaluation was classified as early skeletal muscle mass depletion (ESMMD).

In addition, the quality of skeletal muscle at the time of the diagnosis was examined by calculating the mean attenuation (MA) of skeletal muscle and the intramuscular adipose tissue content (IMAC) of paraspinal muscles.

Therefore, only for baseline CT scans, MA, i.e., density of the skeletal muscle tissue was measured in HU and IMAC was calculated according to literature by dividing CT density of the multifidus muscles (HU) with CT density of subcutaneous fat (HU).

Higher IMAC indicates a greater content of adipose tissue in muscle and, consequently, suggests a lower skeletal muscle quality [18,19].

We used specific cut-off values for SMI, MA, SFI and VFI. We used these cut-off values in accordance with their prognostic role highlighted in two large cohorts reported by Martin et al. and Ebady et al. [14,19]. Sarcopenia was defined as SMI < 43 cm^2^/m^2^ in male patients with BMI < 25 kg/m^2^ and SMI < 53 cm^2^/m^2^ if BMI > 25 kg/m^2^; in female patients, sarcopenia was set at SMI < 41 kg/m^2^ irrespective of BMI. Cut-off values for MA were <41 HU in non-overweight patients (BMI < 25 kg/m^2^) and <33 HU if BMI > 25 kg/m^2^ for both sexes. Sarcopenic obesity was defined as sarcopenia combined with overweight or obesity (BMI > 25 kg/m^2^). The cut off values for VFI, SFI and TAI were 52.9 cm^2^/m^2^ in males and 51.5 cm^2^/m^2^ in females, 50 cm^2^/m^2^ in males and 42 cm^2^/m^2^ in females and 107.7 cm^2^/m^2^ in males and 102.2 cm^2^/m^2^ in females, respectively [14,20].

### 2.2. Statistical Analysis

Data on baseline characteristics and body composition are shown as mean and SD. The median overall survival (OS) and progression free survival (PFS) were determined using the Kaplan–Meier method. Differences in demographic and clinical data between groups were evaluated using the Fisher exact test for categorical variables and independent *t*-test for continuous variables. The best cut-off for laboratory values were defined by ROC curve distribution.

Cox proportional hazards regression model was used to determine the relationship of explanatory variables to survival as hazard ratios (HR) and 95% confidence intervals (CI). Logistic regression was used to describe and explain the relationship between dependent binary variables and independent variables. Odds ratio (OR) together with 95% confidence interval (CI) were provided for logistic regression analyses. Independent variable statistically significant in the univariate analyses were used to build the multivariate analysis. All tests were 2-sided and *p* < 0.05 was considered statistically significant.

MedCalc package (MedCalc1 version 16.8.4) was used for all statistical analyses.

## 3. Results

### 3.1. Patients Characteristics

The present study included 40 patients with a confirmed diagnosis of advanced gastric adenocarcinoma treated with first-line chemotherapy between November 2015 to December 2019 in Modena Cancer Center. The main characteristics of patients enrolled in the study are summarized in Table 1. Overall, 82.5% of patients were younger than 70 years and the 60% of patients were male. The ECOG performance status was 0–1 in the 87.5% of patients at baseline. Since we considered patients with advanced disease, in our sample, only six patients (15%) were submitted to a previous gastrectomy. The first line regimens were mainly doublet chemotherapy with fluoropirimidin and platinum-derivative: 26 patients performed folfox (5-fluorouracil + oxaliplatin), four patients the TOGA regimen (cisplatin + 5-fluorouracil + trastuzumab), four patients a triplete regimen with EOX (epirubicin + oxaliplatin + capecitabine), three patients Xelox (capecitabine + oxaliplatin), and three patients monotherapy with 5-fluorouracil as De Gramont regimen.

The median duration of first line chemotherapy was 6 months (range 1.68–16.38 months); in 7 of 40 (17%) patients an early discontinuation of the treatment was required due to toxicities or worsening of patient clinical conditions.

The mainly reported toxicity was blood count alteration with neutropenia and anemia in 11 patients (27%); afterward, gastrointestinal alterations (mainly nausea and vomit) and peripheral neuropathy in 8 patients (19%). The grade of these adverse events was not reported in our clinical records but none of these was a grade 4. Overall, 20 patients (50%) were reported to receive a second line therapy, consisting prevalently in the association ramucirumab and paclitaxel accordi2ng to the guidelines. In particular, 8/20 (40%) patients in the first group and 12/20 (60%) patients in second group were treated with a second line therapy.

Concerning the anthropometric characteristics, median BMI of the entire sample was 23.59 kg/m^2^. The prevalence of baseline sarcopenia and sarcopenic obesity were 42.5% (17/40) and 7% (3/40), respectively.

### 3.2. Prognostic Factors

The first part of our analysis was addressed to research clinical and anthropometric prognostic parameters and the correlation between these variables and clinical benefit in the whole sample.

Overall, after a median follow-up of 16.4 months, mOS was 12.07 months, whereas mPFS was 6.18 months.

All covariates retrieved were tested within a univariate model.

We evaluated the prognostic impact of baseline clinical, laboratory and anthropometric measures finding a significant interaction between ECOG (ECOG 2 vs. ECOG 0/1. HR 12.74; 95% C.I. 0.66 to 243.85, *p* < 0.001), LMR (LMR > 2.1 vs. LMR < 2.1. HR 3.47; 95% C.I. 1.35 to 8.91, *p* 0.0095), PCR (PCR > 2.2 vs. PCR < 2.2. HR 3.71; 95% C.I. 1.38 to 9.93, *p* 0.009), PNI (PNI > 38.6 vs. PNI < 38.6. HR 3.58; 95% C.I. 1.36 to 9.42, *p* 0.009) and overall survival. Concerning the anthropometric parameters, only ESMMD > 10% from baseline to the first radiological revaluation significantly impact on mOS (HR 2.57, 95% CI 1.13–5.82, *p* = 0.0023) (Table 2).

**Figure 2:**
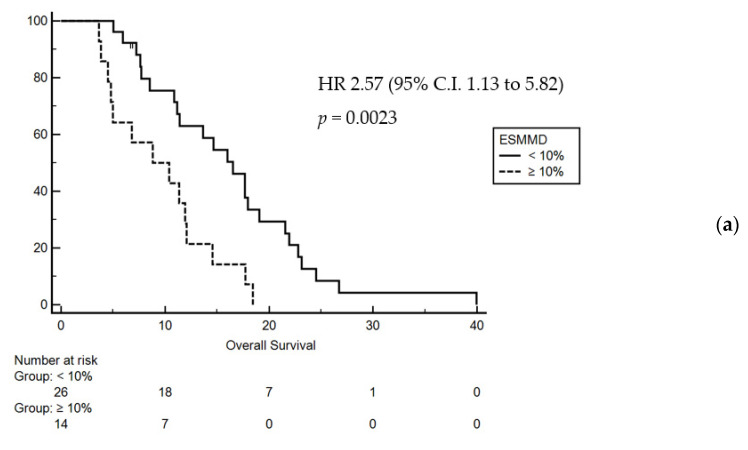

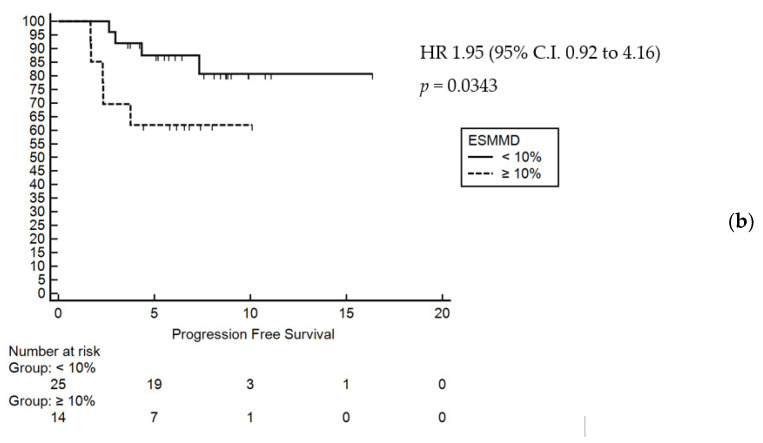
Kaplan–Meier curves for overall survival (**a**) and progression free survival (**b**) according to early skeletal muscle mass depletion (ESMMD). HR: Hazard Ration; CI: Confidence Interval.

Following adjustment for significantly prognostic covariates at univariate analysis, a multivariate analysis was performed, which confirmed ECOG PS (0–1 vs. 2 HR 49.32, 95% CI 7.32–331.95, *p* = 0.0001) and ESMMD > 10% (HR 2.47 95% CI 1.05–7.09, *p* = 0.0375) as the only independent prognostic factors in terms of OS and PFS (Figure 2).

Moreover, by performing a logistic regression analysis, ECOG PS was highlighted to be the only clinical parameter correlated with clinical benefit (defined as stable disease and/or partial response vs. progression disease) (OR 7.25, 95% C.I. 0.9876 to 53.2239, *p* = 0.0004).

Overall, no correlation has been highlighted between anthropometric parameters and toxicities from treatment.

On the other hand, a relationship has been reported between several clinical and bio-humoral variables and radiological assessment of early variation of SMI. In particular, our analysis confirmed a strong link between ESMMD and baseline LDH > 460 U/l (OR 7.91; 95% CI 1.31–47.51, *p* = 0.0046), baseline CRP > 2.2 mg/dL (OR 20.0; 95% CI 1.65–241.73, *p* = 0.006) and weight decrease during treatment (OR 0.82; 95% C.I. 0.71 to 0.94, *p* = 0.0009).

### 3.3. Role of Nutritional Assessment

The second part of our analysis was focused on the role of an early and scheduled nutritional evaluation. We searched for differences in terms of clinical, anthropometric and survival outcomes between the first group of patients which received a nutritional evaluation at oncology’s discretion and the second group of patients which received a standardized nutritional evaluation at the baseline and then every 2–4 weeks during treatment. Overall, 20 patients were included in the first group of patients and 20 patients were included in the second group of patients.

Clinical characteristics were well balanced between the two groups of patients as shown in Table 3.

No significant differences in terms of OS and PFS were reported between the two groups of patients; in return, significative differences in adipose tissue modification during treatment have been highlighted. In particular, our analysis showed that patients reserved to a nutritional scheduled support experienced a mean gain in subcutaneous fat (SFI) of 11.4% at the first radiological evaluation vs. baseline; contrarily, patients reserved to an occasional nutritional support experienced a mean lost in SFI of 3.97% at the first radiological evaluation vs. baseline. Consistently, we demonstrated in patients submitted to a scheduled nutritional support a mean gain in visceral fat of 8.55% at the first radiological evaluation vs. baseline, whereas in patients without a scheduled nutritional evaluation, we found a mean loss of visceral fat of 10.21% at the first revaluation vs. baseline. No significant differences in median ESMMD (−7.52% vs.−2.94%) or median ∆BMI (−3.7% vs.−1.7%) were reported in the two groups of patients.

## 4. Discussion

Recently, the role of sarcopenia in gastric cancer has been focused among perioperative setting. Preoperative muscle mass quality and malnutrition are strictly related to higher surgical risk and delayed and prematurely interrupted adjuvant treatment [21]. Few data are available on the prognostic role of malnutrition in mGC patients [22,23].

In this study, we evaluated a small cohort of 40 advanced GC patients treated with first line palliative chemotherapy in Modena Cancer Center between 2016 and 2019. We evaluated the prognostic impact of baseline clinical, laboratory and anthropometric measures finding a significant interaction between ECOG (ECOG 0/1 vs. ECOG 2, *p* < 0.001), LMR (LMR > 2.1 vs. LMR < 2.1, *p* 0.0095), PCR (PCR > 2.2 vs. PCR < 2.2, *p* 0.009), PNI (PNI > 38.6 vs. PNI < 38.6, *p* 0.009) and overall survival.

In our group of patients, the prevalence of baseline sarcopenia and sarcopenic obesity were 42.5% (17/40) and 7% (3/40) but neither baseline sarcopenia nor BMI at diagnosis significantly affected survival. Conversely, ESMMD > 10% (reported in 14/40 patients) and sarcopenia at first re-evaluation (reported in 18/40 patients) during palliative chemotherapy were associated with shorter mPFS and mOS. In multivariate analysis, in addition to ECOG performance status, only early SMI reduction >10% was statistically relevant and associated with both worse PFS and OS (median PFS 7.3 months vs. 4.4 months, *p* 0.038; median OS 16.5 months vs. 8.5 months, *p* 0.0375). The relevant role of ECOG performance status was highlighted also for its significant impact on clinical benefit at first evaluation (OR 7.25; 95% C.I. 0.9876 to 53.2239; *p* 0.049).

The prevailing role of ESMMD over baseline sarcopenia in metastatic setting has been previously reported not only for gastric cancer patients [20] but also in patients with different gastrointestinal cancers [24,25,26,27]. In the IMPACT trial, in particular, in patients with metastatic pancreatic cancer, ESMMD ≥ 10% was significantly associated with worse OS (HR: 2.16; 95% CI 1.23–3.78; *p* = 0.007) and PFS (HR: 2.31; 95% CI 1.30–4.09; *p* = 0.004) [27].

Beside the role of SMI depletion, another point of our study was the evaluation of the prognostic impact of muscle tissue quality (IMAC and MA) as well as early changes in subcutaneous and visceral adipose tissue during chemotherapy, but none of these parameters were clinically relevant maybe due to the small sample size of the study. The prognostic role of muscle quality at baseline is reported in other setting [19,27,28,29,30,31] but in a recent Dutch series enrolled 88 mGC patients treated with capecitabine and oxaliplatin as first line chemotherapy, no correlation between muscle attenuation and survival was confirmed [24].

Several studies confirmed a well-known association between muscle mass depletion and systemic inflammation [21,32,33]. Systemic inflammation has been reported as a strong prognostic factor in cancer progression. In patients with cancer, pro-inflammatory mediators cause an energetic imbalance between catabolic and anabolic pathways. IL-6, IL-2, IL-10, epidermal growth factor (EGF) and IFN exert their effect by activating the signal transducer and activator of transcription 3 (STAT3) producing loss of muscle mass. The levels of IL-6, TNFa and CRP have been reported to be significantly up-regulated in sarcopenia patients. The activation of a pro-inflammatory status can also lead to insulin resistance and muscle depletion through the activation of the ubiquitin-proteasome proteolytic pathway [34]. In our series, we confirmed the strong link between early muscle mass loss and systemic inflammatory indexes because we noted that baseline LDH > 460, CRP > 2.2 and weight decrease during treatment significantly predicted ESMMD.

The second part of our work was an exploratory analysis on the role of a scheduled nutritional evaluation.

As we know, the nutritional support should be considered as an integrated treatment in patients receiving palliative anti-cancer treatment. Patients identified by screening for unintentional weight loss in last 3–6 years or decreased oral intake, should receive adequate nutritional counselling and support but few studies reported a significative benefit of nutritional support in advanced cancer patients [12,17,35,36].

In our center from 2018, all patients with mGC were evaluated through MUST screening test and then taking charge from a nutritional perspective. They performed a nutritional evaluation every 2–4 weeks alongside the scheduled oncological examination. Before 2018, the nutritional path was at the discretion of oncologists and often the first nutritional evaluation was delayed.

Twenty patients were diagnosed in 2016–2017 and subjected to an occasional involvement of the nutritional team; conversely, the other group of patients were evaluated at diagnosis and then every 2 or 4 weeks with scheduled nutritional visit. No significant difference in OS and PFS were reported but relevant difference in adipose tissue modification has been found.

About subcutaneous fat, a mean gain of 15.38% of SFI vs. baseline SFI was reported between patients with nutritional scheduled support (+11.4% at 3 months vs. baseline) vs. patients with occasional nutritional evaluation (−3.97% at 3 months vs. baseline). Concurrently, a relevant mean gain of 18.76% was reported about the visceral fat between patients in the first group (+8.55% vs. baseline) vs. patients in the latter one (−10.21% vs. baseline).

No relevant difference in median ESMMD (−7.52% vs. −2.94%) or median ΔBMI (−3.7% vs. −1.7%) were reported between the 2 groups.

The prognostic role of adipose tissue in gastrointestinal cancers is still debating [37]. In patients with pancreatic and biliary carcinoma underwent resection, high VSR (visceral to subcutaneous ratio) was reported as a negative prognostic factor in a single Japanese series [38,39]. In a classic trial, enrolled patients underwent gastrectomy followed by adjuvant chemotherapy, a marked loss of visceral or subcutaneous fat significantly predicted shorter DFS and OS [40].

In a large series of 1473 gastrointestinal and respiratory metastatic cancer patients, low TAI (total adipose index) was associated with increased mortality (mOS 19.8 months vs. 14.0 months) [19]. In metastatic colorectal cancer, higher VFI has been associated with shorter OS in patients treated with chemotherapy and anti-VEGF antibody but not in those treated with chemotherapy alone [41]. In a post hoc analysis of two non-randomized phase II trials in the same setting, low SFI (HR 1.63; 1.23–2.17) and low VFI (HR 1.48; 1.09–2.02) were associated with an increased risk of dying confirming the protective role of obesity [42].

In addition, in our series, this protective role was confirmed although not statistically significative maybe due to the small sample size: patients with a higher baseline SFI (>50 cm^2^/m^2^ in males and > 42 cm^2^/m^2^ in females) experienced an increased median overall survival (13.65 vs. 11.94 months), such as patients with a higher VFI (>52.9 cm^2^/m^2^ in males and >51.5 cm^2^/m^2^ in females; mOS 13.65 vs. 11.34 months). Moreover, patients with VFI gain during chemotherapy experienced an increased median overall survival (16.02 months vs. 11.18 months), such as patients with SFI gain (14.64 months vs. 11.18 months) and patients with TAI gain (13.65 months vs. 11.35 months).

Our analysis has several limitations: firstly, the limited number of patients and the retrospective design of the trial. Secondly, we reported a number of patients treated with a consequent second line therapy, which could have influenced the survival outcomes of the two groups of patients considered. Thirdly, patients without available CT scans or lost at follow up were excluded from this trial leading to a possible selection bias. Moreover, we could not have a comprehensive report of the relation between body composition parameters and chemotherapy toxicities or quality of life due to few medical records about these items and the retrospective nature of the study.

## 5. Conclusions

In conclusion, as reported in other metastatic setting, we confirm the prognostic impact of ESMMD > 10% during the first 3 months of first line chemotherapy in metastatic gastric cancer. The impact of ESMMD > 10% is independent from weight loss and could be predicted by some immune-inflammatory markers such as CRP and LDH at baseline.

The prognostic role of a scheduled nutritional assessment could not be highlighted, perhaps due to the sample size of this series, but a relevant gain in adipose tissue (SFI, VFI, TAI) is reported in this group of patients, suggesting clinical benefit due to the protective role of obesity in metastatic gastrointestinal cancers; therefore, a scheduled nutritional assessment and intervention should be evaluated in metastatic gastric cancer patients. However, the role of this approach deserves further confirmation in large prospective trials.

## Figures and Tables

**Table 1:** Patients’ characteristics.

Variable	N(%)
Age	
<70 years	33 (82.5%)
≥70 years	7 (17.5%)
Gender	
Male	24 (60.0%)
Female	16 (40.0%)
Site of primary tumor	
Gastroesophageal junction	5 (12.5%)
Fundus	3 (7.5%)
Body	12 (30.0%)
Fundus and body	4 (10.0%)
GE junction, fundus and body	1 (2.5%)
Antrum	7 (17.5%)
Body and antrum	6 (15.0%)
Diffuse/linitis	2 (5.0%)
Previous gastrectomy	
Yes	6 (15.0%)
No	34 (85%)
Eastern Cooperative Oncology Group Performace Status (ECOG PS) at 1^line chemotherapy start	
0–1	35 (87.5%)
≥2	5 (12.5%)
N° of metastatic sites at 1^line chemotherapy start	
1	12 (30.0%)
≥2	27 (67.5%)
Unknown	1 (2.5%)
Metastatic site at 1^line chemotherapy start	
Liver	5 (12.5%)
Nodes	11 (27.5%)
Peritoneum	24 (60.0%)
Lung	4 (10.0%)
Bone	11 (27.5%)
Others	5 (12.5%)
Body mass index—BMI (kg/m^2^)	
<25	28 (70.0%)
≥25	12 (30.0%)
Prognostic nutritional index—PNI	
<38.6	8 (20.0%)
≥38.6	18 (45.0%)
Unknown	14 (35.0%)
Type of first line treatment	
Single agent	2 (5.0%)
Combination	38 (95.0%)
Laboratory parameter at first-line chemotherapy start	
Neutrophil-lymphocyte Ratio—NLR (Mean ± standard deviation)	5.1 ± 3.5
<4.8	17 (42.5%)
≥4.8	11 (27.5%)
Unknown	12 (30.0%)
Platelet-lymphocyte ratio—PLR (Mean ± standard deviation)	260.8 ± 127.1
<217	12 (30.0%)
≥217	16 (40.0%)
Unknown	12 (30.0%)
Lymphocyte-monocyte ratio—LMR (Mean ± standard deviation)	3.0 ± 1.4
≤2.1	7 (17.5%)
>2.1	21 (52.5%)
Unknown	12 (30.0%)
Systemic Immune-Inflammation Index (SII) (Mean ± standard deviation) (×10^3^ cells/µL	1465 ± 1041
≤1110	20 (30.0%)
>1110	16 (40.0%)
Unknown	12 (30.0%)
Albumine (Mean ± standard deviation) (g/dL)	3.6 ± 0.5
≤3.5	19 (47.5%)
>3.5	17 (42.5%)
Unknown	4 (10.0%)
C reactive Protein—PCR (Mean ± standard deviation) (mg/dL)	3,2 ± 4,6
<2.2	13 (32.5%)
≥2.2	8 (20.0%)
Unknown	19 (47.5%)
Carcinoembryonic antigen—CEA (Mean ± standard deviation) (ng/mL)	201.6 ± 683.2
≤5	23 (57.5%)
>5	14 (35.0%)
Unknown	3 (7.5%)
Carbohydrate antigen 19.9—Ca 19.9 (Mean ± standard deviation) (U/mL)	622 ± 1856
≤37	20 (50.0%)
>37	17 (42.5%)
Unknown	3 (7.5%)

**Table 2:** Uni and multivariate analysis for overall survival.

	Univariate		Multivariate
	HR (95% CI)	*p*	
Age (≥65 vs. <65 years)	1.08 (0.57–2.07)	0.8110	
ECOG PS (2 vs. 0–1)	12.75 (0.67–243.86)	<0.0001	0.0001
Site of M (>1 vs. 1)	1.92 (0.90–4.11)	0.058	
NLR (≥4.8 vs. <4.8)	2.00 (0.84–4.75)	0.0711	
LMR (<2.1 vs. ≥2.1)	3.03 (0.88–10.46)	0.0060	
PCR (≥2.2 vs. <2.2 mg/dL)	3.1 (0.98–9.78)	0.0055	
CEA (≥5 vs. <5 ng/mL)	0.67 (0.33 to 1.36)	0.2763	
PNI (≥38.6 vs. <38.6)	0.34 (0.11–1.06)	0.0058	
SII (≥1110 vs. <1110 (×10^3^ cells/µL)	0.95 (0.44–2.07)	0.9104	
BMI (<25 vs. ≥25 kg/m^2^)	0.79 (0–37–1.70)	0.5215	
IMAC (≤−0.33 vs. >−0.33)	0.67 (0.34–1.32)	0.2233	
VFI (≥52.9 cm^2^/m^2^ in males and 51.5 cm^2^/m^2^ in females vs. <52.9 cm^2^/m^2^ in males and 51.5 cm^2^/m^2^ in females)	1.24 (0.66–2.36)	0.5010	
SFI (≤50 cm^2^/m^2^ in males and 42 cm^2^/m^2^ in females vs. >50 cm^2^/m^2^ in males and 42 cm^2^/m^2^ in females)	0.76 (0.40–1.43)	0.3921	
Sarcopenia sec. Martin (Yes vs. No)	1.40 (0.72–2.73)	0.3058	
Sarcopenia at revaluation sec. Martin (Yes vs. No)	2.24 (1.07–4.69	0.0117	
ESMMD (≥10% vs. <10%)	2.57 (1.13–5.83)	0.0036	0.0375

**Table 3:** Patients categorized according to the nutritional approach.

	Occasional Nutritional Evaluation (*n* = 20)	Systematic Nutritional Evaluation (*n* = 20)	*p*
Age			
<70 years	16 (80%)	17 (85%)	1.00
≥70 years	4 (20%)	3 (15%)	
Gender			
Male	13 (65%)	11 (55%)	0.54
Female	7 (35%)	9 (45%)	
Site of primary tumor			
Gastroesophageal junction	4 (20%)	1 (5%)	0.2334
Fundus	3 (15%)	0	
Body	4 (20%)	8 (40%)	
Fundus and body	2 (10%)	2 (10%)	
GE junction, fundus and body	1 (5%)	0	
Antrum	3 (15%)	4 (20%)	
Body and antrum	3 (15%)	3 (15%)	
Diffuse/linitis	0	2 (10%)	
Previous gastrectomy			
Yes	3 (15%)	3 (15%)	
No	17 (85%)	17 (85%)	
ECOG PS at 1^line chemotherapy start			
0–1	19 (95%)	16 (80%)	0.3416
≥2	1 (5%)	4 (20%)	
N° of metastatic sites at 1^line chemotherapy start			
1	7 (35%)	5 (25%)	0.7311
≥2	13 (65%)	14 (70%)	
Unknown	0	1 (5%)	
Metastatic site at 1^line chemotherapy start			
Liver	1 (5%)	4 (20%)	0.1173
Nodes	8 (40%)	3 (15%)	
Peritoneum	11 (55%)	13 (65%)	
Lung	1 (5%)	3 (15%)	
Bone	8 (40%)	3 (15%)	
Others	4 (20%)	1 (5%)	
Body mass index—BMI (kg/m^2^)			
<25	14 (70%)	14 (70%)	1.00
≥25	6 (30%)	6 (30%)	
Prognostic nutritional index—PNI			
<38.6	2 (10%)	6 (30%)	0.2077
≥38.6	9 (45%)	9 (45%)	
Unknown	9 (45%)	5 (25%)	
Type of first line treatment			
Single agent	1 (5%)	1 (5%)	1.00
Combination	19 (95%)	19 (95%)	
Laboratory parameter at first-line chemotherapy start			
Neutrophil-lymphocyte Ratio— NLR (Mean ± standard deviation)	4.6 ± 1.9	5.5 ± 4.2	
<4.8	7 (35%)	10 (50%)	0.3765
≥4.8	5 (25%)	6 (30%)	
Unknown	8 (40%)	4 (20%)	
Platelet-lymphocyte ratio— PLR (Mean ± standard deviation)	240.4 ± 81.9	276.1 ± 150.6	
<217	5 (25%)	7 (35%)	0.3835
≥217	7 (35%)	9 (45%)	
Unknown	8 (40%)	4 (20%)	
Lymphocyte-monocyte ratio— LMR (Mean ± standard deviation)	2.8 ± 1.4	3.1 ± 1.5	
≤2.1	3 (15%)	4 (20%)	0.3858
>2.1	9 (45%)	12 (60%)	
Unknown	8 (40%)	4 (20%)	
Lactate dehydrogenase— LDH (Mean ± standard deviation) (U/L)	631 ± 460	505 ± 661	
≤460	8 (40%)	15 (75%)	0.0769
>460	6 (30%)	2 (10%)	
Unknown	6 (30%)	3 (15%)	
Albumine (Mean ± standard deviation) (g/dL)	3,7 ± 0,5	3,4 ± 0,5	
≤ 3.5	8 (40%)	11 (55%)	0.6056
> 3.5	10 (50%)	7 (35%)	
Unknown	2 (10%)	2 (10%)	
C reactive Protein— PCR (Mean ± standard deviation) (mg/dL)	3.7 ± 5.2	3.0 ± 4.2	
<2.2	4 (20%)	9 (45%)	0.0820
≥2.2	3 (15%)	5 (25%)	
Unknown	13 (65%)	6 (30%)	
Carcinoembryonic antigen— CEA (Mean ± standard deviation) (ng/mL)	178.5 ± 537.3	216.2 ± 744.4	
≤5	11 (55%)	12 (60%)	0.8283
>5	7 (35%)	7 (35%)	
Unknown	2 (10%)	1 (5%)	
Carbohydrate antigen 19.9— Ca 19.9 (Mean ± standard deviation) (U/mL)	646.7 ± 1013.6	604.9 ± 2217.4	
≤37	9 (45%)	11 (55%)	0.529
>37	9 (45%)	8 (40%)	
Unknown	2 (10%)	1 (5%)	

## Data Availability

Data available on request from the authors.
